# The Jena Voice Learning and Memory Test (JVLMT): A standardized tool for assessing the ability to learn and recognize voices

**DOI:** 10.3758/s13428-022-01818-3

**Published:** 2022-06-01

**Authors:** Denise Humble, Stefan R. Schweinberger, Axel Mayer, Tim L. Jesgarzewsky, Christian Dobel, Romi Zäske

**Affiliations:** 1 Department of Experimental Otorhinolaryngology, Jena University Hospital, Stoystrasse 3, 07743 Jena, Germany; 2 Department for General Psychology and Cognitive Neuroscience, Institute of Psychology, Friedrich Schiller University Jena, Am Steiger 3/1, 07743 Jena, Germany; 3 Department of Psychological Methods and Evaluation, Institute of Psychology, Institute of Psychology and Sports Science, University of Bielefeld, Universitätsstr. 25, 33615 Bielefeld, Germany

**Keywords:** Voice recognition, Individual differences, Phonagnosia, IRT

## Abstract

The ability to recognize someone’s voice spans a broad spectrum with phonagnosia on the low end and super-recognition at the high end. Yet there is no standardized test to measure an individual’s ability of learning and recognizing newly learned voices with samples of speech-like phonetic variability. We have developed the Jena Voice Learning and Memory Test (JVLMT), a 22-min test based on item response theory and applicable across languages. The JVLMT consists of three phases in which participants (1) become familiarized with eight speakers, (2) revise the learned voices, and (3) perform a 3AFC recognition task, using pseudo-sentences devoid of semantic content. Acoustic (dis)similarity analyses were used to create items with various levels of difficulty. Test scores are based on 22 items which had been selected and validated based on two online studies with 232 and 454 participants, respectively. Mean accuracy in the JVLMT is 0.51 (SD = .18) with an empirical (marginal) reliability of 0.66. Correlational analyses showed high and moderate convergent validity with the Bangor Voice Matching Test (BVMT) and Glasgow Voice Memory Test (GVMT), respectively, and high discriminant validity with a digit span test. Four participants with potential super recognition abilities and seven participants with potential phonagnosia were identified who performed at least 2 SDs above or below the mean, respectively. The JVLMT is a promising research and diagnostic screening tool to detect both impairments in voice recognition and super-recognition abilities.

**Supplementary Information:**

The online version contains supplementary material available at 10.3758/s13428-022-01818-3.

## Introduction

Vocal communication involves more than the exchange of linguistic information. Non-linguistic vocal information allows us, for example, to recognize with whom we are interacting, which is particularly relevant when visual information is unavailable. Voice recognition abilities can be considered a key prerequisite for successful social interactions and are present remarkably early in life, even in utero (DeCasper & Fifer, 1980; Kisilevsky et al., 2003). Since no utterance can be repeated in exactly the same way twice, the challenge of voice recognition is “to generalize across variability within a speaker, and to distinguish within speaker variability from systematic between-speaker variability” (Schweinberger et al., 2014). Successful voice learning is therefore characterized by the establishment of relatively abstract, speech-invariant representations of a speaker’s voice in long-term memory. This means that speaker recognition should be possible from novel (previously unheard) speech content (Zäske et al., 2014), including a foreign language, pseudo-speech or even non-speech utterances (Skuk & Schweinberger, 2013). Although we know that this remarkable ability varies considerably between people, there are hardly any standardized tools to assess human voice recognition. In particular, we are lacking tests that capture speech-invariant voice recognition for complex utterances with high phonetic variability (i.e., sentences, rather than syllables or isolated vowels). We are providing the scientific community with a novel test to fill this gap (available online from: https://osf.io/cyr23/).

The voice signal is rich in information (Schweinberger et al., 2014), which can be categorized into acoustic (formant, jitter etc.), linguistic (speech content), rapidly changing para-linguistic (emotion), as well as more stable extra-linguistic components (age, gender, personality characteristics, and identity). Functional models of person perception (e.g., Belin et al., 2004; Young et al., 2020) illustrate how, when, and by means of which brain areas perceivers process this information. An important assumption of these models is that both familiar voices and faces can be recognized despite considerable low-level variability within the same person. In the case of voices, this variability may be introduced by speech prosody or content, as well as filters (e.g., telephone) or noisy backgrounds. Faces can change dramatically in appearance depending on viewpoint, lighting, or expression. Yet we can typically recognize familiar people across different utterances and images. This is achieved by robust long-term representations, so-called voice and face recognition units (VRUs and FRUs, respectively). These recognition units are acquired during familiarization with a new person and hold abstract and idiosyncratic information about each voice and face we know. In the case of voices, identification may be achieved by any acoustic cue which is typical for a given speaker (Schweinberger et al., 2014). Note that according to our definition of voice memory, VRUs may therefore store information related to vocal cues that are determined by both, anatomical inevitabilities (e.g., individual voice timbre, f0), and learned cues (e.g., dialects, prosody), provided that these cues are distinctive and characteristic for a given speaker.

While both voices and faces serve to recognize people, research has focused mainly on faces. Evidence for large individual differences in face perception abilities has challenged the notion that humans are face experts by default (Diamond & Carey, 1986). As a consequence, several paradigms and tests have been developed from the 1980s (e.g., BFMT; Benton et al., 1983; RMT; Warrington, 1984) until today, to assess these differences. Existing tests can be broadly set into three categories: 1) face perception tests using discrimination/matching/sorting tasks for unfamiliar faces (e.g., GFMT; Burton et al., 2010; FICST; Jenkins et al., 2011; Stacchi et al., 2020; OFMT; Stantic et al., 2022), 2) face recognition tests on familiar faces (e.g., YBT; Bruck et al., 1991; BTWF; Russell et al., 2009; Stacchi et al., 2020) and 3) tests assessing learning and recognition abilities for previously unfamiliar faces (e.g., CFMT; Duchaine & Nakayama, 2006; CFMT + ; Russell et al., 2009). These tests are being continuously developed further, often emerging initially as experimental paradigms, before they were validated with large samples of participants and thus transformed into standardized tests (Jenkins et al., 2011; Stacchi et al., 2020). Some tests have been normed for special groups (e.g., children, CFMT-C; Croydon et al., 2014), including people with exceptional face recognition skills (super-recognizers; CFMT + ; Russell et al., 2009). Individual differences in face perception abilities have been linked to several phenomena including autism, social anxiety, empathy, prosopagnosia, or super-recognition skills that may promote eyewitness identification of criminals among the police (reviewed in Wilmer, 2017).

Although individual differences in voice perception skills are relevant for analogous fields, voice tests are still relatively scarce, as specified further below. It seems clear that some of these differences relate to the listener's age and gender. For instance, Zäske et al. (2018) found an advantage of young vs. old adult listeners to recognize newly learned voices, and Skuk and Schweinberger (2013) found that female listeners outperform male listeners when recognizing voices of personally familiar speakers. Irrespective of the age and gender of listeners, voice recognition can span a broad ability spectrum from phonagnosia, i.e., the inability to recognize familiar speakers by their voices (Van Lancker & Canter, 1982), to super-recognition (Aglieri et al., 2017). Various efforts have been made to better understand how voice recognition abilities dissociate from other auditory abilities (Peretz et al., 1994; Van Lancker & Kreiman, 1986, 1987) and to assess the prevalence of voice recognition impairments using celebrity voices (Quaranta et al., 2016; Shilowich & Biederman, 2016). Roswandowitz et al. (2014) screened 1,057 individuals and identified two cases of phonagnosia, by means of tailor-made ad hoc voice tests. This has been a common approach in case studies on voice perception (also cf. Garrido et al., 2009) and due to the lack of standardized tests at the time. While voice researchers across the world still use various paradigms and speech materials, standardized and validated tests of voice perception and memory would not only make research more comparable across labs and disciplines. They would likely also stimulate research on exceptional voice processing abilities and prompt advancements in basic research and applied fields analogous to face tests. Ultimately, valid and reliable tests form the foundation for common international standards and test batteries to assess person recognition abilities across labs (compare Ramon, 2021).

Voice and face processing skills serve similar functions in social interactions, share at least some processing mechanisms, and vary considerably among the population, with congenital impairments and super-recognition skills often going unnoticed. We therefore anticipate an increasing demand for standardized voice tests, particularly when considering that voice perception research has taken off only relatively recently (Belin et al., 2004) and is largely oriented at the face literature. Most of the currently available voice tests target the processing of affective vocal information (Bänziger et al., 2009; Nowicki & Duke, 1994; Scherer & Scherer, 2011; Schlegel & Scherer, 2016; Schlegel et al., 2014), while only two tests are concerned with voice identity processing (Aglieri et al., 2017; Mühl et al., 2018).

The Bangor Voice Matching Test (BVMT; Mühl et al., 2018) measures voice *perception* abilities, i.e., matching of unfamiliar voices, while the Glasgow Voice Memory Test (GVMT; Aglieri et al., 2017) assesses *recognition* memory for newly learned voices. The BVMT requires participants to make “same/different”-judgments for 80 randomly presented voice pairs uttering short syllables (female and male voice pairs presented block-wise). The authors based their test on item response theory (IRT) in a two-step-selection and validation process. This was to achieve adequate variations in item difficulty and discrimination and to account for the aforementioned broad spectrum of voice recognition ability. Participants’ mean performance was 84.6% (SD = 7.2; range, 61.3–97.5%) and test–retest reliability of this 10-min self-paced test is high (*r* = 0.86). Note, that the ability to match voices presented in immediate succession is typically considered an ability separate from general voice recognition (Van Lancker et al., 1985; von Kriegstein & Giraud, 2004) and relies on short-term memory for *unfamiliar* voices.

By contrast, we aim at developing a memory test which involves the study of voices across several repetitions and their subsequent recognition from long-term memory, similar to the GVMT. The GVMT is a 5-min screening tool asking participants to learn eight speakers (four female) who utter the French vowel /a/ three times. Directly following, participants perform an “old/new” recognition task on eight novel and eight learned speakers by means of the same vowel stimuli as used during learning trials. This procedure is then repeated with bell sounds serving as a control condition. Mean performance was slightly better for bells (M = 85.6%) than for voices (M = 78.8%). Concerning reliability of the voice test, inter-rater reliability was moderate (ICC coefficient = 0.38) and reported internal consistency was excellent (Cronbach’s alpha = 0.9973). Note that Aglieri et al. (2017) chose to use simple vowel stimuli (rather than more complex utterances) in their test, and presented the same voice samples at test as during learning. While this approach of measuring memory for a particular and rather simple voice sample may be a sensible first step, we considered that the logical next steps would be to use more complex utterances, and to change speech content between study and test. This was in order to capture two important properties of everyday voice memory: First, we usually communicate with *complex* utterances, typically sentences, whose phonetic variability and suprasegmental information has been shown to benefit speaker recognition relative to shorter utterances and isolated vowels (Schweinberger & Zäske, 2019; Schweinberger et al., 1997). Second, by changing speech content between study and test we aim at capturing the acquisition of relatively abstract voice identity representations in semantic memory (e.g., Belin et al., 2004; Young et al., 2020), rather than memory for a specific study episode involving a particular voice sample. Crucially, if learning leads to the formation of VRUs, these should allow for speaker recognition across a range of utterances, including novel (non-studied) utterances (Sheffert et al., 2002; Zäske et al., 2014). Accordingly, we considered the ability to extract idiosyncratic voice patterns from complex speech and the generalization to novel utterances to be core functions and markers of successful voice learning, which we wanted to capture with the present test. The Jena Voice Learning and Memory Test (JVLMT) thereby puts a different focus on voice learning and recognition than the GVMT.

With the JVLMT, we aimed at constructing a versatile tool to measure voice memory abilities across a considerable ability spectrum in a time-efficient manner, and largely independent of language backgrounds of users and listeners. As the main differences to the GVMT, we use sentence stimuli (rather than vowels) and ask listeners to recognize newly learned speakers from *novel* utterances (rather than repeating study samples), in order to increase ecological validity of the test. By using pseudo-speech rather than a particular existing language we aimed to minimize possible advantages in speaker recognition due to spurious language-familiarity effects (Fleming et al., 2014; Levi, 2019; Perrachione & Wong, 2007; Perrachione et al., 2011; Zarate et al., 2015). However, because even pseudo-speech has to be based on the phonology some language, we preferred English as an international language. We thereby intend to make the test compatible with as many as possible applications and research projects worldwide, considering that the most influential research on person perception originates from English-speaking countries, as evidenced by contributions in current compendia (Calder et al., 2011; Frühholz & Belin, 2019; Kreiman & van Lancker Sidtis, 2011). As speakers we invited volunteers, capable of pronouncing our pseudo-sentences according to English pronunciation rules. The focus here was on obtaining fairly standardized speech material to mimic phonetic variability in existing languages, such that certain speakers would not stand out in the learning phase, just because they used fundamentally different pronunciations of the same pseudo-words. In the course of item selection, the eight learning (target) speakers happened to be German native speakers with English as L2. Listeners were international participants who were required to understand English to ensure that the written instructions to the test would be understood by all participants. The language background of our participants was otherwise irrelevant as a criterion for participation.

As discussed above, the JVLMT provides a novel and ecologically valid tool, which complements the existing GVMT and BVMT in order to assess individual differences in voice learning and memory skills. We anticipate that several basic and applied fields will benefit from the JVLMT. Regarding basic research, the JVLMT may promote better understanding of the psychological, neurophysiological or linguistic mechanisms that are related to individual voice recognition skills. Moreover, the JVLMT could become a useful tool to forensic phonetics, to the extent that relationships between test results and the reliability of earwitness testimony can be demonstrated. In the context of the healthcare system, the JVLMT may help identify voice perception deficits and (potentially) their recovery in neurological or hearing-impaired patients, as well as in individuals with autism. Finally, the JVLMT may be employed to detect voice recognition experts whose work may help to increase public security.

With a testing time of ~ 22 min, the JVLMT presents an efficient research tool to determine individual voice memory abilities within the normal population, and offers an initial screening for possible exceptional voice memory abilities (phonagnosia or super-recognition). The test consists of a learning phase, a repetition phase and a testing phase and is structurally based on the Cambridge Face Memory Test (CFMT, Duchaine & Nakayama, 2006): At first, participants are trained to become familiar with 8 voice identities in two learning blocks. Each learning trial consists of a familiarization part as well as an immediate recognition part, where the studied target speaker has to be recognized among two unfamiliar foils. Before the testing phase, participants revise all studied voice identities in a repetition phase. In the testing phase, participants then perform a three-alternative forced-choice-task (3AFC) in a closed set to detect the voice identities they had previously studied. The process of item-selection was based on IRT (Eid & Schmidt, 2014; Embretson & Reise, 2013) to ensure that the JVLMT contains items which cover a considerable ability spectrum. Items were selected in a two-fold process. The first phase (see Item selection) encompasses the initial (item-selection) version of the JVLMT with 72 items. Through item-response-analysis, we selected 26 items conforming to the Rasch model (for details, see Results). In a second phase (see Test validation), this 26-item validation version of the JVLMT was validated with two convergent validation tests, the GVMT (Aglieri et al., 2017) and the BVMT (Mühl et al., 2018) as well as a discriminant validation test assessing auditory working memory (Digit Span Test, DS, as adapted from Della Sala et al., 2010). Finally, we performed another item-analysis and selected the final 22 test items conforming to the Rasch model. Please see the left panel of Fig. 2 for an overview of development steps.

## Methods & results

All described studies were conducted in accordance with the Declaration of Helsinki and were approved by the Faculty Ethics Committee of the Friedrich Schiller University of Jena (FSV 18/33).

### Test development

#### Speech material

Our aim was to develop a test which uses utterances containing relatively natural and sentence-like suprasegmental information, yet would not contain recognizable speech content, and would thus establish relatively fair conditions for listeners of various language backgrounds. We implemented the construction of fifty pseudo-sentences through Wuggy, a pseudo-speech generator based on written input (Keuleers & Brysbaert, 2010). Wuggy generates pseudo-words within the phonotactic restrictions of a given input language. As input, we used the most frequent English nouns, verbs, and adjectives, as based on subtitles of films from Brysbaert and New (2009). Specifically, we used “contextual diversity” as a measure for frequency, i.e., the number of films with subtitles containing a given word in lower-case. We selected the 100 most frequent nouns, and the 50 most frequent verbs and adjectives, respectively, to design six word-sentences with a sentence-like syntax (article-noun–verb-article-adjective-noun).^1^ Note that these input “sentences” were semantically meaningless, despite containing meaningful words (e.g., “the safety deserve they hungry stomach”). The output were several versions of pseudo-sentences per input sentence (e.g., “ble sulpty debepts thek henbly stopapt” to keep with the present example). Please consult supplemental materials Sect. 2.1 (available from: https://osf.io/cyr23/) for further selection criteria and a complete list of the final 50 pseudo-sentences (Table S1).

#### Speakers

##### Selection criteria

Speakers were recruited via posters and flyers around the university campus, university-based mailing lists and social media. Eligible speakers were between 18 and 35 years old, and reported no vocal pathologies. Note that our criterion for inclusion was profound English pronunciation skills for pseudo-sentences, rather than English as a native language. For judging pronunciation skills for pseudo-sentences, we requested three self-recorded sample sentences (“hig etoa beseng thek fanoes ligs”, “ble loff disbiss ble tommible pollion”, “thek truft explect heg usfious rour”). Based on these audio samples, the principal investigator selected and invited speakers for the voice recordings if they had an authentic “th” and round “r” sound, but no prominent accent. In preparation for the recording and to standardize pronunciation, speakers were sent a 30-min video tutorial containing all pseudo-sentences in written form along with audio speech samples as uttered by an American native speaker.

##### Sample characteristics

We recorded *N* = 80 speakers (66 students, 50% identifying as male and female, respectively) with a mean age of M_age_ = 24.2 (SD_age_ = 3.8). Speakers reported the following native language(s): German (*n* = 63), English (*n* = 7), Turkish (*n* = 4), Spanish (*n* = 2), English and Chinese, Persian, Russian and Ukrainian (*n* = 1 respectively). Speakers indicated their individual level of acquired foreign language proficiency on a five-point scale from 1—“fluent” to 5—“not at all fluent”. Of the 72 non-native speakers of English, 41 evaluated their English language level with "1”, 25 with “2” and 6 with “3″. Fifty-seven of our speakers have always been non-smokers, 13 identified as smokers or having smoked before, and ten speakers did not respond to this question.

##### Recording and processing procedure

The recording session lasted for approximately 2 h. After giving written informed consent, speakers were recorded in a quiet and anechoic room using a Sennheiser MD 421-II microphone with pop protection and a Zoom H4n audio interface (16-bit resolution, 48-kHz sampling rate, mono) using a fixed and standardized protocol for the 50 pseudo-sentences. Before a sentence was recorded, the sample sentence from the pronunciation tutorial was presented once via loudspeakers along with the written sentences on a laptop in front of the speakers. Each sentence was typically recorded three times or until the experimenter was satisfied with the utterance. Final stimuli were recordings with clear pronunciation, the least artifacts as well as the least background noise. Stimuli were processed using PRAAT software (Boersma & Weenink, 2001), and cut to contain only the utterance. Utterances were then RMS normalized to 60 dB. Overall, our database for stimulus selection contains 4000 utterances (50 pseudo-sentences from 80 speakers).

### Stimulus selection and item development

The recognition test of the JVLMT is a 3AFC task with a closed set of one target voice and two foils per test trial. To vary task difficulty, we manipulated acoustic similarity between the three test voices of a given trial, henceforth called “voice triplets”. This was achieved by choosing voice triplets based on their relative location in a 3D acoustic space, according to their F0, formant dispersion (FD; mean frequency difference of successive formants 1 through 4) and harmonics-to-noise ratio (HNR). The notion that acoustic similarity between voices may affect accuracy in a 3AFC- voice recognition task, is derived from evidence that the location of voices in this space is related to perceptual measures including distinctiveness ratings (Latinus et al., 2013; Zäske et al., 2020) and voice discriminability (Mühl et al., 2018). Based on this reasoning we first selected only triplets spanning equilateral triangles in the voice space, i.e., three voices with equal distances to one another. This was to ensure that, within a given triplet and recognition trial, no single voice would stand out perceptually and would thus be equally confusable with any of the other two voices (cf. also the target-nontarget-similarity-theory by Duncan and Humphreys, (1989)). Second, to vary task difficulty across trials we selected triplets spanning small, medium-sized or large equilateral triangles (cf. supplemental materials for further details on item selection).

**Fig. 1:**
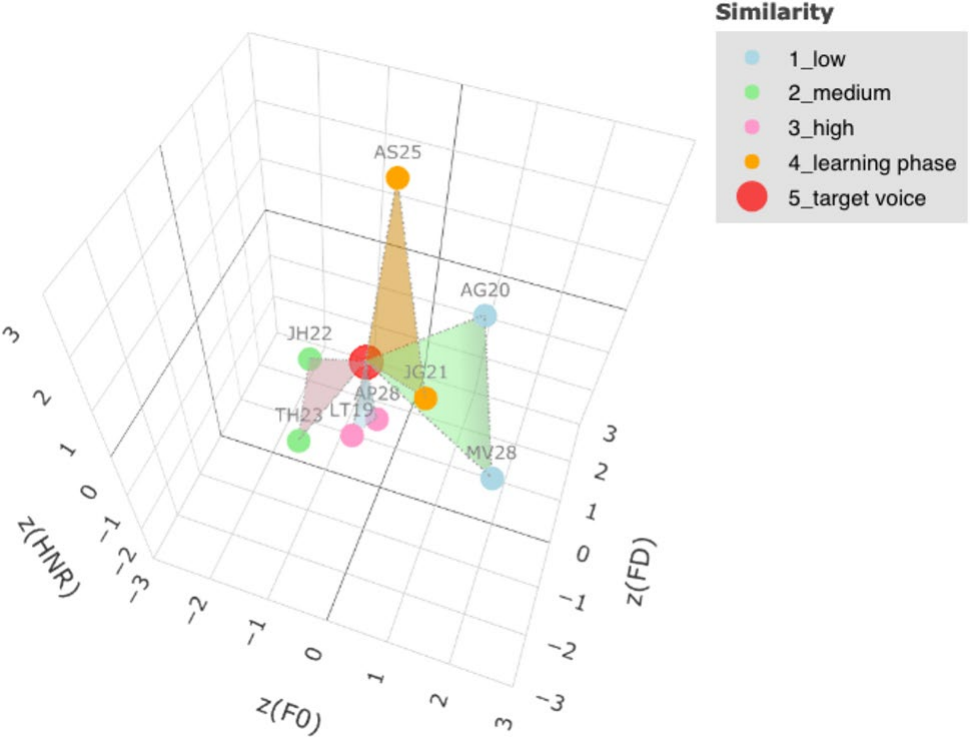
Acoustic voice space containing all voice triplets of an exemplary male learning voice identity who is here represented as “target voice”. *Colored triangles* represent different levels of acoustic similarity between the target and two respective foils, as used on a given test trial. Triplets used for immediate recognition trials in the learning phase had medium acoustic similarity

As learning voices (and subsequent target voices) we determined the four male and female voice identities which were part of most triplets. This was to enhance possible combinations with foils in test trials. Thus, the remaining 36 voices of each gender served as foils in the 3AFC tasks. For each learning identity we then selected one triplet per similarity level to be used in test trials, and one additional triplet of medium difficulty for the immediate recognition trials of the learning phase. We ensured that all foils were only part of one triplet, such that foils for one learning identity were never presented with another learning identity. At test, each triplet was presented at three presentation durations (250 ms, 750 ms, and full-length).^2^ Figure 1 shows a voice space with exemplary triplets and levels of acoustic similarity.

The 50 pseudo-sentences available for each speaker were randomly assigned to learning and recognition trials, avoiding sentence repetitions between the learning and testing phase. This was to capture voice recognition that generalizes to new speech contents, rather than reflecting mere stimulus recognition. To exclude presentation order as a cue to target identity, we also balanced the position of target and foils within 3AFC trials.

### General paradigm and design

**Fig. 2:**
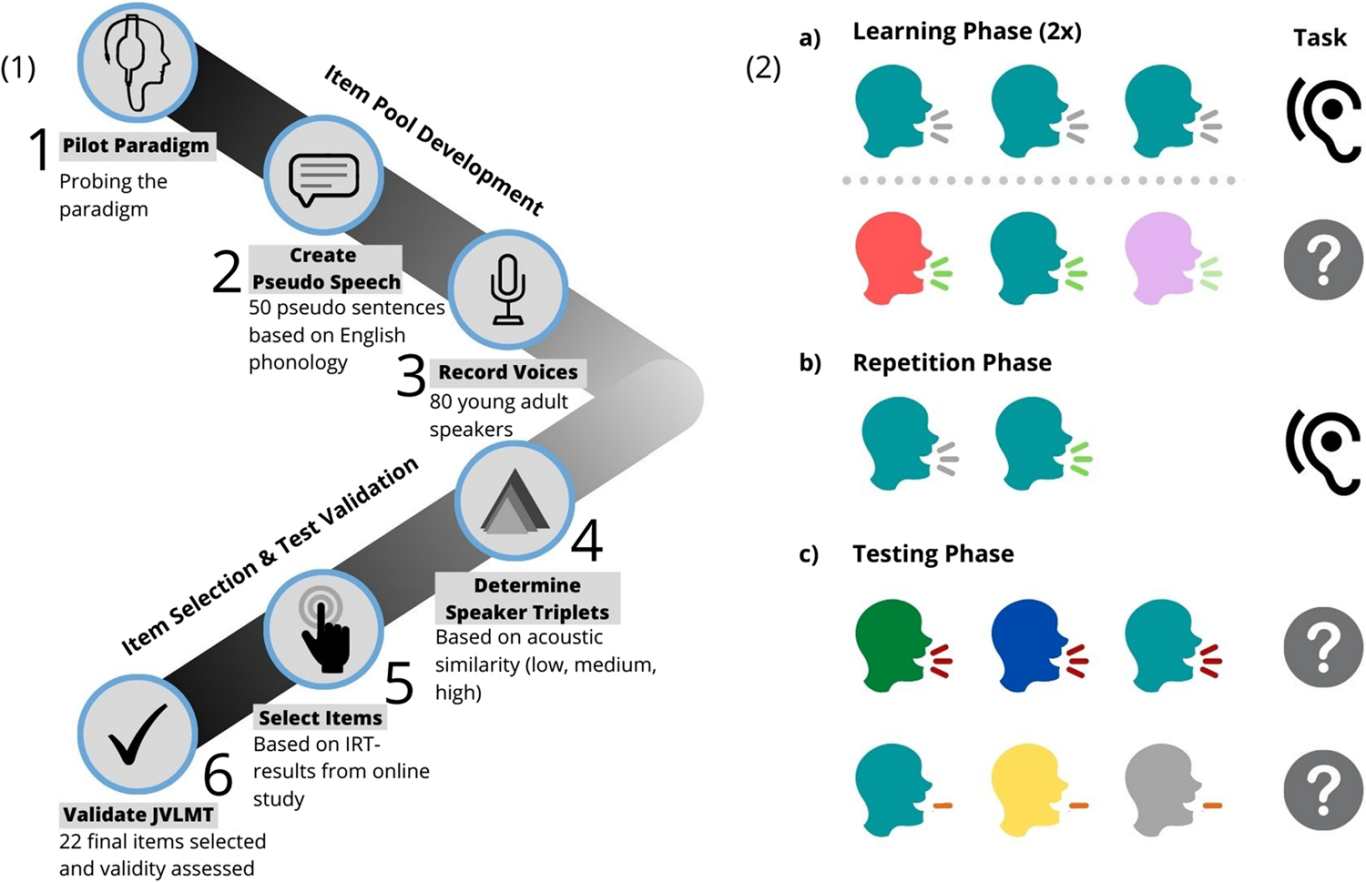
Overview of the development steps (1) and the JVLMT paradigm (2**a–c**) as exemplified for one learning voice identity (shown in *teal*). The JVLMT consists of three phases: The learning phase (**a**) consists of a familiarization trial followed by an immediate recognition trial comprising the learned target voice and two foils (shown in *red* and *pink*). In the repetition phase (**b**), participants listen to the learning voice identities who repeat a sentence from the familiarization trial and from the immediate recognition trial of the learning phase. In the testing phase (**c**), participants perform a 3AFC-task in which they have to recognize the previously learned voice identities among two new foils (shown in *green* and *blue*). Duration of utterances varied (i.e., 250 ms, 750 ms, full duration) between trials as symbolized by the number of speech output lines. Speech content varied between familiarization and test in the learning phase and also between the learning and testing phase as represented by varying colors of speech output lines. The figure was designed with Canva (Canva, 2020; Gehred, 2020)

The JVLMT is divided into three phases: learning, repetition and testing. While the learning and testing phases require participants to enter responses, the repetition phase is a passive listening task. The right panel (2) of Fig. 2 gives an overview of the general paradigm, as used for the initial item-selection version, the validation version and the final version of the JVLMT. The detailed trial timings and the chronological order of stimuli as appearing in the final JVLMT along with acoustic measures can be found in the supplemental materials (Fig. S1 and Table S9, respectively). In the learning phase, participants are familiarized with each of the eight learning voice identities and tested for immediate recognition abilities. On each familiarization trial they hear three different sentences by one learning speaker whose voice they are asked to memorize for an ensuing test. On the immediately following test trial, they hear a previously unheard sentence, uttered once by each of three consecutive speakers. Participants are asked to select the learned target speaker among the two foils, before the next learning speaker is presented and so forth. While sentence contents in the learning trials are identical across learning speakers, sentence content varies across immediate recognition trials. After all eight voices have been learned and tested once, this procedure is repeated in a second learning-test-cycle, with the only difference that new sentences are used on immediate recognition trials.^3^ This is to introduce more phonetic variability and thereby facilitate voice learning. Thus, by the end of the learning phase, each learning speaker has been presented with five different sentences (for a chronological overview of stimuli as appearing in the final JVLMT, cf. Table S9).

In the repetition phase, participants passively listen to two consecutive sentences from each of the eight learning voice identities again (one sentence, respectively taken from the familiarization and immediate recognition trials of the learning phase).

The testing phase consists of 3AFC-trials in which participants have to recognize the eight learning identities among two foils, respectively. The number of recognition trials is 72 for the item selection phase, 26 for the test validation phase, and 22 for the final test. Note that none of the foils from the learning phase was used during the testing phase, thus all foils were indeed unfamiliar voice identities. Similarly, utterances in the testing phase had never been heard before to provide a measure for voice learning proper, rather than for mere stimulus recognition. To further increase variability of item difficulty at test (cf. Schweinberger et al., 1997), we presented targets and foils at three durations (250 ms, 750 ms, or full length, measured from voice onset).

Sample size estimations for item selection and test validation were based on Charter (1999, 2003).

### Item selection

#### Listeners

We analyzed datasets obtained online from *N* = 232 healthy participants (*n* = 101, *n* = 130 and *n* = 1 identifying as female, male, and other, respectively) from the general population worldwide who confirmed normal hearing abilities, absence of neurological or psychological disorders, and with no reported use of psychoactive substances. Participants were 18 to 72 years old (*M* = 37.4, *SD* = 11.5 years). The great majority were from the USA (*n* = 165), followed by India (*n* = 58), with English being the most prevalent native language (*n* = 211, including multilinguals). Most participants reported using English at least frequently in a passive way (reading and/or listening; *n* = 228) and in an active way (speaking, writing; *n* = 225) every day. None of the participants were familiar with any of the speakers prior to the experiment. In case of impaired vision, participants were instructed to use glasses or contact lenses. Participation was rewarded with $7/h. Before the study began participants gave informed consent via ticking the respective box. Overall, 107 further participants were excluded because they did not complete the experiment entirely, reported neurological or psychiatric disorders, indicated technical difficulties or had more than 10% of omission errors. A detailed overview of reasons for exclusion can be found in the supplementary materials (Table S2).

#### Stimuli and materials

As a basis for item selection the initial version of the JVLMT comprised 288 experimental stimuli. These were presented in full length on familiarization trials, and with various durations on test trials (250 ms, 750 ms, or full length) to vary task difficulty (cf. 2.3 General Paradigm & Design). Seventy-two stimuli were presented in the learning phase (8 learning voices × 5 sentences, 16 foils × 2 sentences) and testing phase (8 learning voices × 3 sentences × 3 durations), respectively. Eighteen further stimuli, not used in the main test, were used in practice and dummy trials preceding the learning phase (*N* = 12) and testing phase (*N* = 6), respectively. Mean stimulus duration for full length experimental stimuli was 3706 ms (SD = 499 ms, range, 2486–5603 ms). A simple beep (100 ms) was used to signal the upcoming end of fixed breaks which we had implemented in the online experiment.

#### Procedure

Programming and data acquisition were done via PsyToolKit (Stoet, 2010, 2017). Recruiting and data collection was done via Amazon’s Mechanical Turk (Buhrmester et al., 2011). Participants were instructed to wear headphones and to complete the study in a quiet environment without background noise using a PC/laptop with a keyboard. They were also asked not to interact with others while completing the study and to stay at the computer during the automated breaks, such that they would not miss the next testing block. Before the experiment participants completed a soundcheck whereby an example stimulus from our database was looped until participants had adjusted the volume to a comfortable hearing level. Following this soundcheck participants started the experiment, which lasted ~ 35 min, including instructions and four fixed breaks of 2 min each, i.e., one break between the repetition and testing phase, and one after every 18 test trials. As a final step, participants filled out a demographic questionnaire. On average, participants needed ~ 51 min to complete the entire study.

##### Learning phase

Participants learned eight randomized voice identities (four female) which had to be recognized among two foils immediately afterwards. Familiarization trials and immediate recognition trials thus alternated until all learning voices had been learned and tested once. Familiarization trials consisted of three different voice samples of the same speaker, prompted by a green circle. On immediate recognition trials the learned speaker identity had to be recognized “as accurately as possible” among three consecutive voices uttering the same, previously unheard sentence. During immediate recognition trials, responses were recorded via number keys (1–3) according to the position of target voice identity in the preceding voice sequence. For responses slower than 4000 ms, participants were prompted to respond faster. For detailed trial procedures and timings, please refer to the supplementary materials (Fig. S1). Participants completed two practice trials before the learning phase. The learning phase lasted ~ 7 min.

##### Repetition phase

Participants passively revised the eight randomized learning voice identities for ~ 2 min. Two previously heard utterances from the learning phase were presented consecutively for each voice identity.

##### Testing phase

Participants completed a 3AFC-task on 72 randomized recognition trials for ~ 23 min. The eight target voice identities from the learning phase had to be recognized in nine trials each. The nine trials contained all possible combinations of the factors ACOUSTIC SIMILARITY (low, medium, high) and DURATION (250 ms, 750 ms, and full-length). As described above, for each target voice identity there was a unique triplet containing two foils for each similarity level. Each triplet was used three times during this testing phase for each level of duration. The trials were structurally equivalent to the immediate recognition trials described above for the learning phase. Please refer to Fig. S1 in the supplementary material for detailed trial sequences for all phases of the JVLMT. Please note, that automated breaks were fixed to two minutes between the repetition and testing phase as well as between every 18 trials of the testing phase. Two beeps signaled the upcoming end of the breaks, i.e., 10 s and 5 s before the experiment continued. Participants completed two dummy trials before the 72 actual trials to prepare them for the short presentation durations. Dummy trials were not analyzed.

#### Results

Data were analyzed using R with the mirt and ez packages (Chalmers, 2012; Fox & Weisberg, 2019; Lawrence, 2016; *R Core Team: The R Project for Statistical Computing*, 2020). Where appropriate, epsilon corrections for heterogeneity of covariances with the Huynh–Feldt method (Huynh & Feldt, 1976) were performed throughout. For all statistical analyses, an alpha-level of *α* = 0.05 was chosen.

##### Performance learning phase

A total of 2.5% of trials were excluded as errors of omission (RT ≥ 4,000 ms). Mean accuracies in the testing phase were significantly greater than chance (> 0.33): *t*(224) = 30.35, *p* < 0.001 (M = 0.70, SEM = 0.01).

##### Performance testing phase

**Fig. 3:**
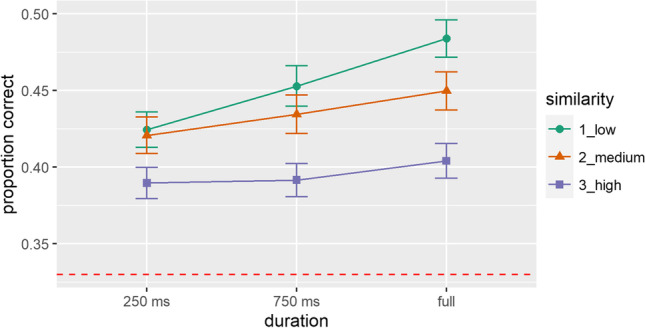
Proportion of correct responses in the testing phase. Depicted factors are *duration* (250 ms, 750 ms, and full length) and *acoustic similarity* (low, medium, and high) of test voices on a given trial. Chance level (0.33) is indicated by the *dotted line*. *Error bars* represent SEMs

A total 1.2% of trials were excluded as errors of omission (RT ≥ 4000 ms). Accuracies were compared with chance in six one-sample *t* tests, separately for all levels of both independent variables (duration: 250 ms, 750 ms and full-length and similarity: low, medium and high). Accuracies were all significantly greater than chance (9.97 ≤ *t*s[695] ≤ 16.83, all *p*s < 0.001): M(SEM)_250 ms_ = 0.41(0.01); M(SEM)_750 ms_ = 0.43(0.01); M(SEM)_full_ = 0.45(0.01); M(SEM)_high_ = 0.40(0.01); M(SEM)_medium_ = 0.44(0.01); M(SEM)_low_ = 0.45(0.01). Please see Fig. 3 for results as well. In a next step, a repeated-measures ANOVA with the factors DURATION (250 ms vs. 750 ms vs. full) and SIMILARITY (low vs. medium vs. high) was calculated based on the performance scores. We found significant main effects (*F*_duration_[2462] = 8.03; *p* < 0.001; η_*p*_^2^ = 0.03); *F*_similarity_[2462] = 18.41; *p* < 0.001; η_*p*_^2^ = 0.07), with no interaction (*F*[4,924] = 1.15; *p* = 0.331; η_*p*_^2^ < 0.01). Main effects were post hoc-tested with adjusted alpha levels for three pairwise comparisons within each factor (alpha level of 0.017). Accordingly, corrected *t* tests revealed significantly higher performance for full length vs. 250 ms durations (*t*[231] = – 3.77; *p* < 0.001). This effect was reduced to a trend for full length vs. 750-ms durations (*t*[231] = – 2.37; *p* = 0.019) and was non-significant for 750-ms vs. 250-ms durations (*t*[231] = – 1.71; *p* = 0.09). For the factor similarity, a significantly higher recognition performance was found for low vs. high (*t*[231] = 6.11; *p* < 0.001), as well as for medium vs. high (*t*[231] = 4.04; *p* < 0.001) voice similarities. The comparison between low and medium similarity did not yield significant differences (*t*[231] = 1.87; *p* = 0.063).

In response to a reviewer request, we also calculated a logistic mixed model to assess possible effects of NATIVE LANGUAGE of the 231 participants whose language background was known (*n* = 211 English vs. *n* = 20 “other”), for details cf. supplemental materials Sect. 2.3 Item selection. Essentially, this analysis yielded no main effect of participants’ NATIVE LANGUAGE at the stage of item selection. We replicated this result for the final 22-item version of the JVLMT, based on a larger participant sample (*N* = 454) of the validation phase (*n* = 407 English native speakers vs. *n* = 47 speakers of “other” native languages). For more details on this analysis, please see supplemental materials (Sect. 2.4 Test validation).

#### Item response analysis

From the initial 72 test items, 26 items conforming to the Rasch model were selected. This means that only items that were in line with assumptions of the Rasch model, were used for the validation version of the JVLMT (26-item JVLMT). These assumptions pertain to Rasch homogeneity and local independence. Specifically, Rasch homogeneity means that all items measure the same underlying construct/ability in the same manner, i.e., the probability of solving an item is a logistic function of the (unidimensional) ability and the item difficulty. Local independence means that a participant’s response to one item should not depend on their response to another item. An important property of the Rasch model, namely, specific objectivity, is that the item difficulties can be separated from the person abilities, meaning that comparisons between persons are invariant over the items used. Similarly, specific objectivity also implies that comparisons between items are invariant across persons, meaning that differences between item difficulties can be generalized beyond the calibration sample. Please note that, in the Rasch model the number of correct responses (as sum score or mean scores) is a sufficient statistic, i.e., it has all the information there is in the data about the ability of the respondent, and can therefore be used as an ability measure. This is not the case in more complex IRT models such as the 2PL or 3 PL models (we refer the interested reader unfamiliar with the approach to Embretson & Reise, 2013).

In order to test the assumptions of the Rasch model for our items, we used likelihood-based fit measures, graphical inspection of empirical item characteristic curves, and analysis of differential item functioning in certain subgroups. Items that seem to violate the assumptions of the Rasch model based on these criteria were classified as ill-fitting and were excluded from the test. An overview of item difficulties and item fit measures of all 26 items using a Rasch model can be found in the supplementary materials (Fig. S2). Note, that in the Rasch model, item difficulty and ability are depicted on the same axis. Consequently, the higher the ability of a person relative to the item difficulty, the higher the probability of solving this item. A person whose ability is equal to the item difficulty has a probability of 0.5 of solving the item.

For the initial 72 items, a Rasch model as well as a 2PL-model was originally calculated.^4^ Model fits were compared using a likelihood ratio test. The fit of the 2PL-model was significantly better (χ^2^ [71] = 258.90, *p* < 0.001), so the item selection-process was started using the 2PL model. In this procedure, ill-fitting items as well as items with a mean accuracy below chance (M_ACC_ < 0.33) were eliminated in a stepwise manner. When 39 items were left, the fit of the 2PL model was no longer significantly better than that of the Rasch model (χ^2^ [38] = 49.21, *p* = 0.105). We then continued the item-selection process based on the Rasch model for reasons of simplicity. Thus, further items were excluded until 28 items conforming to the Rasch model were left. Testing for the possible existence of method factors by comparing multidimensional and unidimensional models did not reveal any significant results. Furthermore, differential item functioning (DIF) of the 28 items was tested for gender and age in two groups each (female vs. male and ≤ 35 years and ≥ 36 years). This analysis revealed some DIF for the two gender groups (*χ*^*2*^ [27] = 56.09, *p* = 0.001). Specifically, two items whose item difficulty parameters were significantly different between the two gender groups were identified and excluded. Therefore, the validation version of the JVLMT contained 26 items with an empirical (marginal) reliability of 0.63. Reliability is defined as the proportion of true score variance relative to the total variance (i.e., true score variance plus error variance). It is a measure that quantifies the measurement accuracy. In the context of our IRT model, we reported the empirical (marginal) reliability (see e.g., Chalmers, 2012). This measure is based on secondary latent trait estimates (obtained using the expected a posteriori (EAP) method) and the corresponding standard errors. For computing the reliability coefficient, the true score variance is estimated by the variance of the secondary latent trait estimates and the error variance is estimated using the average squared standard errors of these estimates. Note that this measure of reliability therefore reflects the mean reliability of all items, i.e., including items with intermediate difficulty and high reliability, as well as easy and hard items with lower reliabilities. A depiction of the item-wise reliability can be seen in the item information curves in the supplementary materials (Fig. S2B).

### Test validation

#### Listeners

We analyzed datasets from *N* = 454 healthy participants (*n* = 156, *n* = 296, *n* = 2 identifying as female, male, or other, respectively) of the general population as obtained online and worldwide. None reported neurological or psychological disorders, or used psychoactive substances. All reported normal hearing abilities and ranged in age from 18 to 74 years with a mean of 37.4 (SD = 12.21). The great majority of participants were from the USA (*n* = 332), followed by India (*n* = 84), with English being the most prevalent native language (*n* = 407, including multilinguals). Self-evaluated language proficiency according to the Common European Framework of Reference for Languages (2001) was mostly “proficient” (C2: *n* = 270; C1: *n* = 78), followed by “upper intermediate” (B2: *n* = 40). Most reported using English at least frequently in a passive way (reading and/or listening; *n* = 439) and in an active way (speaking, writing; *n* = 438). For detailed information on the distribution of native language and language proficiency cf. tables S4 and S5 in the supplemental materials. None of the participants were familiar with any of the speakers prior to the experiment. In case of impaired vision, participants were asked to use their glasses or contact lenses. Participation was rewarded with $7/h. The study started after participants had ticked a box to give their informed consent for participation.

Overall, *k* = 397 participants were excluded because they did not complete all experiments, participated multiple times, reported psychiatric or neurological disorders, indicated technical difficulties or had ≥ 2SD above the mean of errors of omission in at least one of the included tests. Please refer to the supplementary materials for a detailed overview of reasons for exclusion.

#### Stimuli and materials

The validation version of the JVLMT comprised 150 experimental stimuli: 72 in the learning phase and 78 in the testing phase. Eighteen further stimuli were used for practice and dummy trials preceding the learning phase (*N* = 12) and testing phase (*N* = 6), respectively. Mean stimulus duration was 3,727 ms (SD = 505 ms, range, 2487–5603 ms). Otherwise, the structure and procedure of this validation version was equivalent to the item-selection version. Items in all phases were randomized once and then kept in this order for each participant.

In addition to the JVLMT, we added three further tests for validation: an auditory working memory test assessing digit span (Della Sala et al., 2010), a voice discrimination test (BVMT by Mühl et al., 2018), and a voice learning test (GVMT by Aglieri et al., 2017). To match sound pressure levels across the JVLMT and the other three tests, all stimuli from the validation tests were normalized to 60 dB.

For the Digit Span Test (DS) we converted a test by Della Sala et al. (2010) into an online version by recording a female voice uttering 55 digit sequences in in English, at a speed of one digit per second. For each sequence length (from four digits to nine digits) we recorded six stimuli plus one practice stimulus. The sounds were processed analogously to the stimuli used in the JVLMT. On each trial participants listened to one digit sequence, and entered the heard digits in the exact same order via their keyboard. Starting with four digits per sequence, the sequence length increased stepwise after six trials. If participants responded correctly on five out of six trials, the sequences increased by one digit on the following six trials and so on. The test aborted when less than five trials of the same sequence length were correctly answered. Note that the criterion of five out of six correct responses per sequence length makes this version harder compared to conventional versions of digit span assessments, such that we would expect lower digit span scores (compare also Mühl et al., 2018). Test scores represent the maximal length of sequences for which participants reproduced at least five items correctly (i.e., a test score of seven would mean that a participant reproduced at least five of the seven-digit-sequences and of shorter sequences correctly, but was unable to recall more than four items of the eight-digit sequences). Depending on participants’ performance the test lasted ~ 4 min.

The BVMT (Mühl et al., 2018) is a 10-min voice matching test and contains 160 syllable stimuli uttered by 160 unfamiliar speakers. Stimuli are presented pairwise in 80 match and non-match trials (40 male and female trials, respectively). Participants indicate via key press on each trial whether two consecutive utterances were produced by the same speaker.

The GVMT (Aglieri et al., 2017) contained 32 stimuli (16 vowel utterances from eight female and eight male speakers as well as 16 bell sounds) and lasts for ~ 5 min. Participants were familiarized with eight voice identities (four female) by listening to the vowel /a/ three times in a row. Immediately afterwards, participants performed an old/new recognition memory task on the eight learned and eight novel voices, all uttering the vowel from the familiarization phase. The second part of the test had the identical structure, but comprised bell sounds instead of voices.

#### Procedure

The procedure for the JVLMT was equivalent to that in the item-selection phase. However, the number of test items was reduced from 72 to 26 after item selection and test duration was reduced to ~ 22 min (including instructions, ~ 7-min learning phase, ~ 2-min repetition phase, one fixed 2-min break, and ~ 7-min testing phase). After completing the validation version of the JVLMT, participants also completed a Digit Span Test (Della Sala et al., 2010), the BVMT (Mühl et al., 2018) and the GVMT (Aglieri et al., 2017) in this fixed order. Participants had fixed breaks of 1 min or 2 min between tests. Within tests one fixed break was set to 2 min for the JVLMT (after the repetition phase), 2 min for BVMT (after the first matching block), and 1 min for the GVMT (after voice recall). Finally, they filled out a demographic questionnaire. On average, participants needed 65 min to complete the entire study.

#### Results

Data were analyzed using R with the “mirt” package (Chalmers, 2012; Fox & Weisberg, 2019; *R Core Team: The R Project for Statistical Computing*, 2020). Where appropriate, epsilon corrections for heterogeneity of covariances with the Huynh–Feldt method (Huynh & Feldt, 1976) were performed throughout. For all statistical analyses, an alpha-level of α = 0.05 was chosen.

##### Item-response analysis

**Fig. 4:**
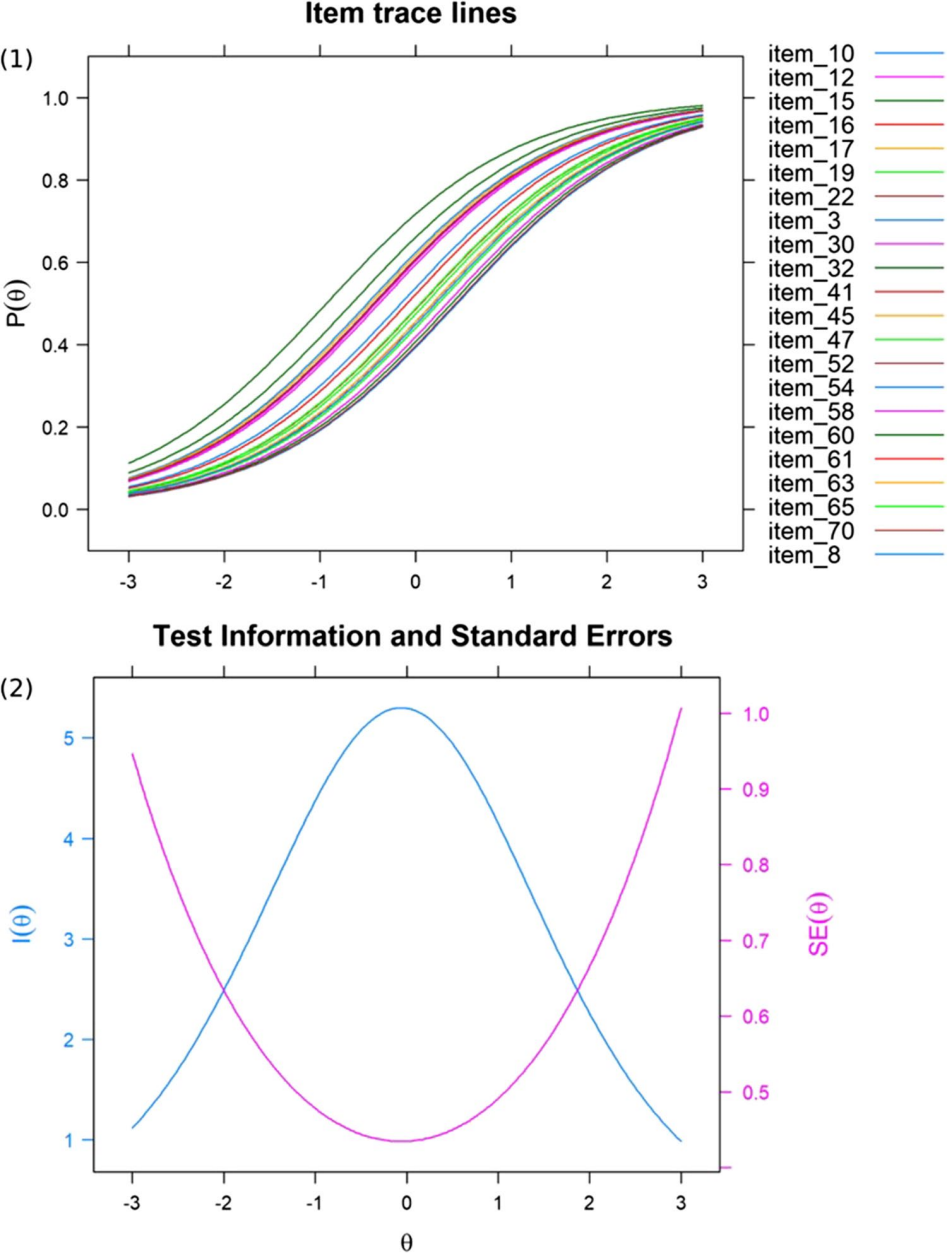
Item-characteristic curves (1) for the 22 items conforming to the Rasch model from the final version of the JVLMT. (2) contains the test information function (*blue line*) and the standard error of ability levels (*pink line*)

Item analyses and final selection was performed using the R-package “mirt” and was based on *N* = 454 datasets obtained online. From the 26 items used in the validation phase, 22 items conforming to the Rasch model were selected for the final version of the JVLMT. Four items of the previous version had to be discarded, because the analysis with the larger dataset revealed that their fit was insufficient. An overview of item difficulties and item fit measures of the final 22 items based on a Rasch model can be found in the supplementary materials (Table S8). A likelihood ratio test indicated a significant better fit of the 2PL model (χ^2^ [21] = 48.58, *p* = 0.001). However, when inspecting the coefficients closer, it became apparent that the Bayesian information criteria (BIC) for the Rasch model (BIC = 13,071.43) was indeed smaller than the BIC for the 2PL-model (BIC = 13,151.34). In line with Preinerstorfer and Formann (2012) this expresses a better model fit for the Rasch model which was therefore selected. Differential item functioning (DIF) of the 22 items was tested for gender and age in two groups each (female vs. male and ≤ 35 years and ≥ 36 years). This analysis revealed no significant DIF. The final version of the JVLMT contains 22 items with an empirical (marginal) reliability of 0.66. Figure 4 contains the item characteristic curves (ICCs) for the final 22 items as well as the test information curve and the standard errors. The following analyses were computed based on performance accuracy for these 22 items.

##### Descriptive results

*Performance learning phase.* We excluded 3.0% as errors of omission (RT ≥ 4000 ms). All responses were significantly greater than chance: *t*(453) = 40.31, *p* < 0.001 (M = 0.69, SD = 0.19).

**Fig. 5:**
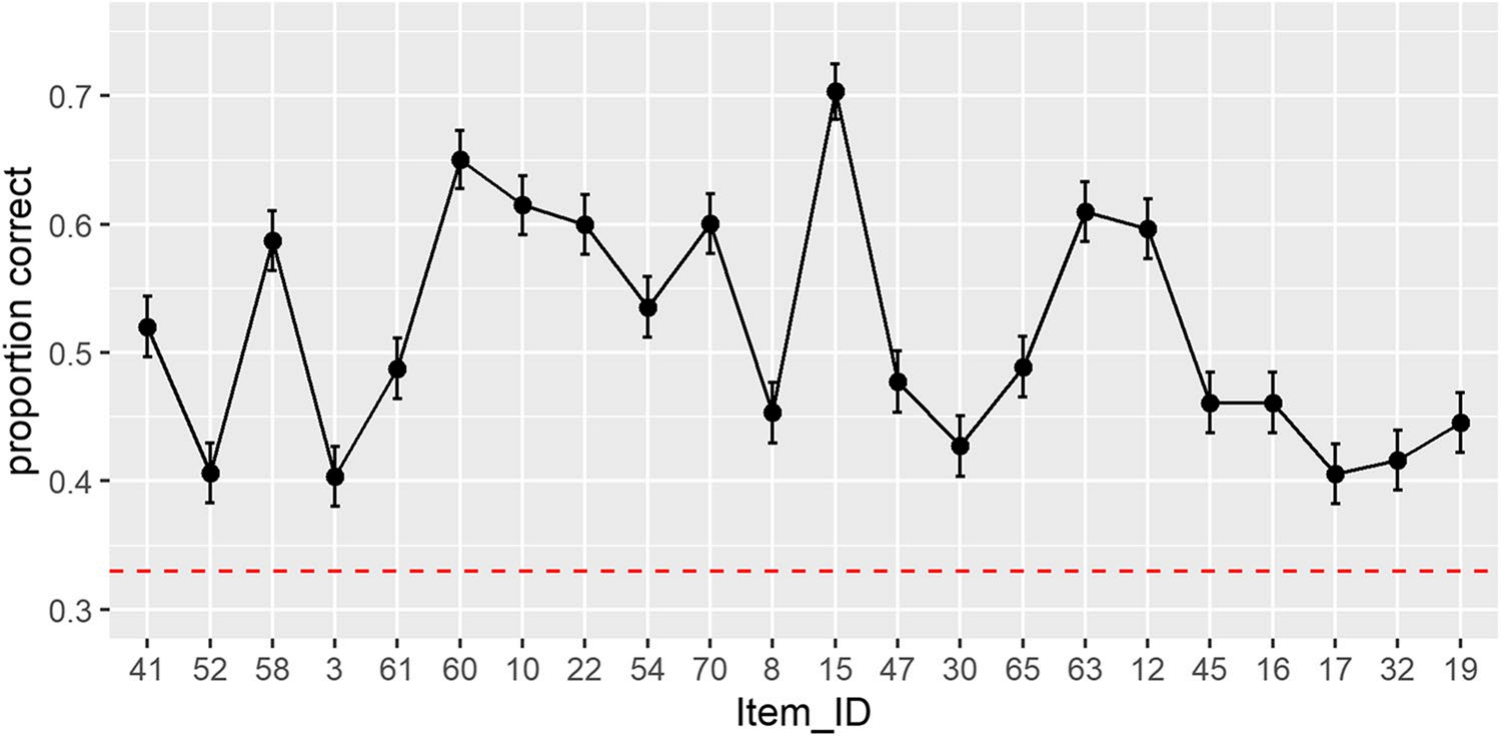
Mean proportion of correct responses for each test item, depicted in chronological order of appearance in the JVLMT, averaged over *N* = 454 online data sets. The *dashed line* represents the chance level (0.33). *Error bars* represent SEMs

**Fig. 6:**
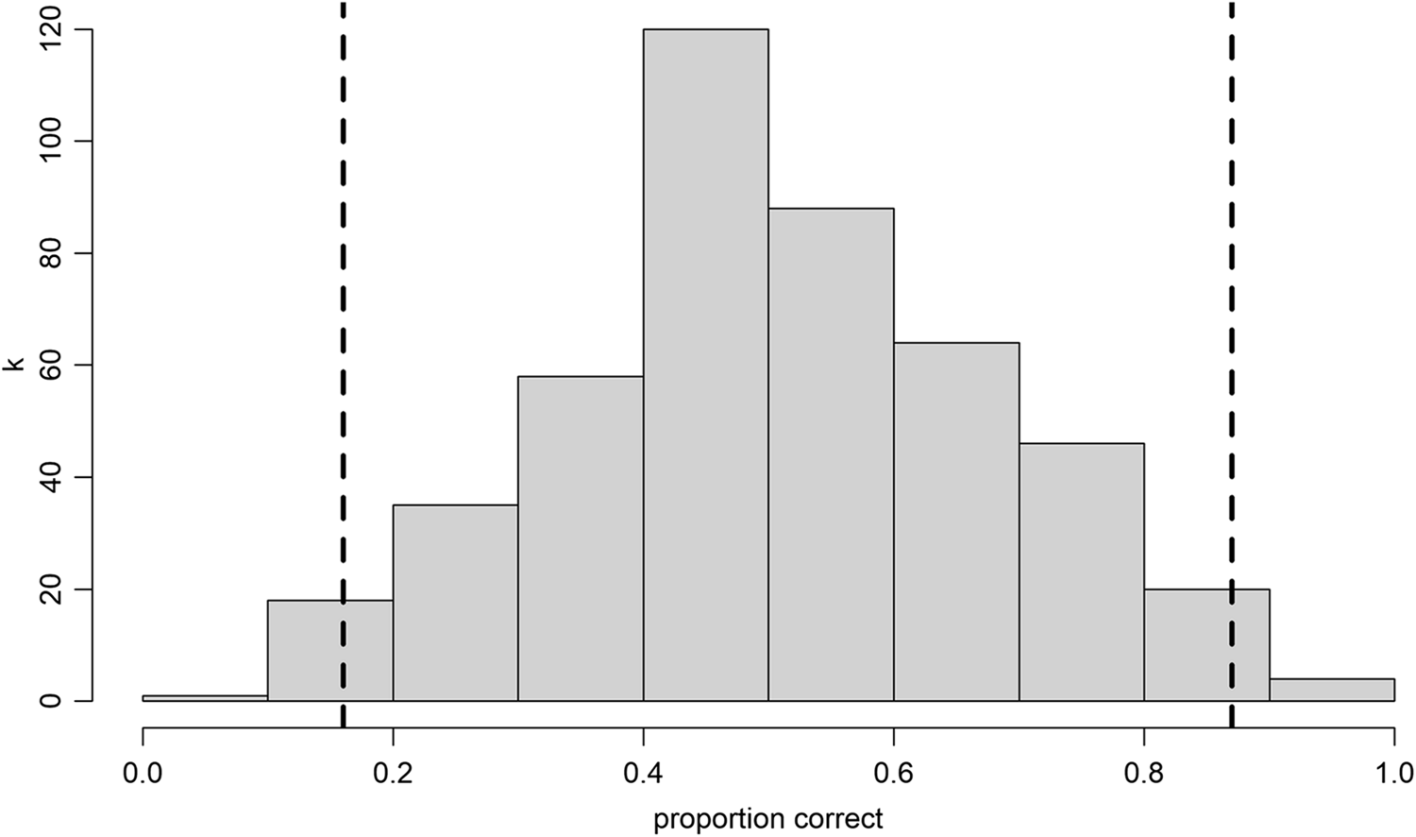
Test score distribution of the JVLMT. *Dashed lines* represent 2 SDs below/above the mean

*Performance testing phase.* Trials with errors of omission (1.7%) were excluded (RT ≥ 4000 ms). Accuracies were significantly above chance: *t*(453) = 21.95, *p* < 0.001 (M = 0.51, SD = 0.18, range, 0.05–0.95). Figure 5 gives an overview of item-wise mean performance. See Fig. 6 for a distribution of mean test scores.

##### Correlations with validation tests

The JVLMT correlated strongly with the BVMT (*r* = 0.56) indicating convergent validity. Correlations to the second convergent validation test, the GVMT, were moderate for bells and voices (*r*_voices_ = 0.36; *r*_bells_ = 0.43). The Digit Span test served as a test for divergent validity. This correlation was weak with *r* = 0.21,^5^ indicating better performance on the JVLMT with increasing digit span. Table 1 shows the descriptive statistics of performance in each of the validation tests.

**Table 1:** Descriptive statistics of validation tests (proportion correct or digit span)

	BVMT (ACC: 0 to 1)	GVMT: voices (ACC: 0 to 1)	GVMT: bells (ACC: 0 to 1)	DS (4 to 9)
M	0.75	0.69	0.77	6
SD	0.14	0.13	0.14	1.32
Range	0.39–0.98	0.31–1	0.31–1	4–9

##### Individual differences

**Fig. 7:**
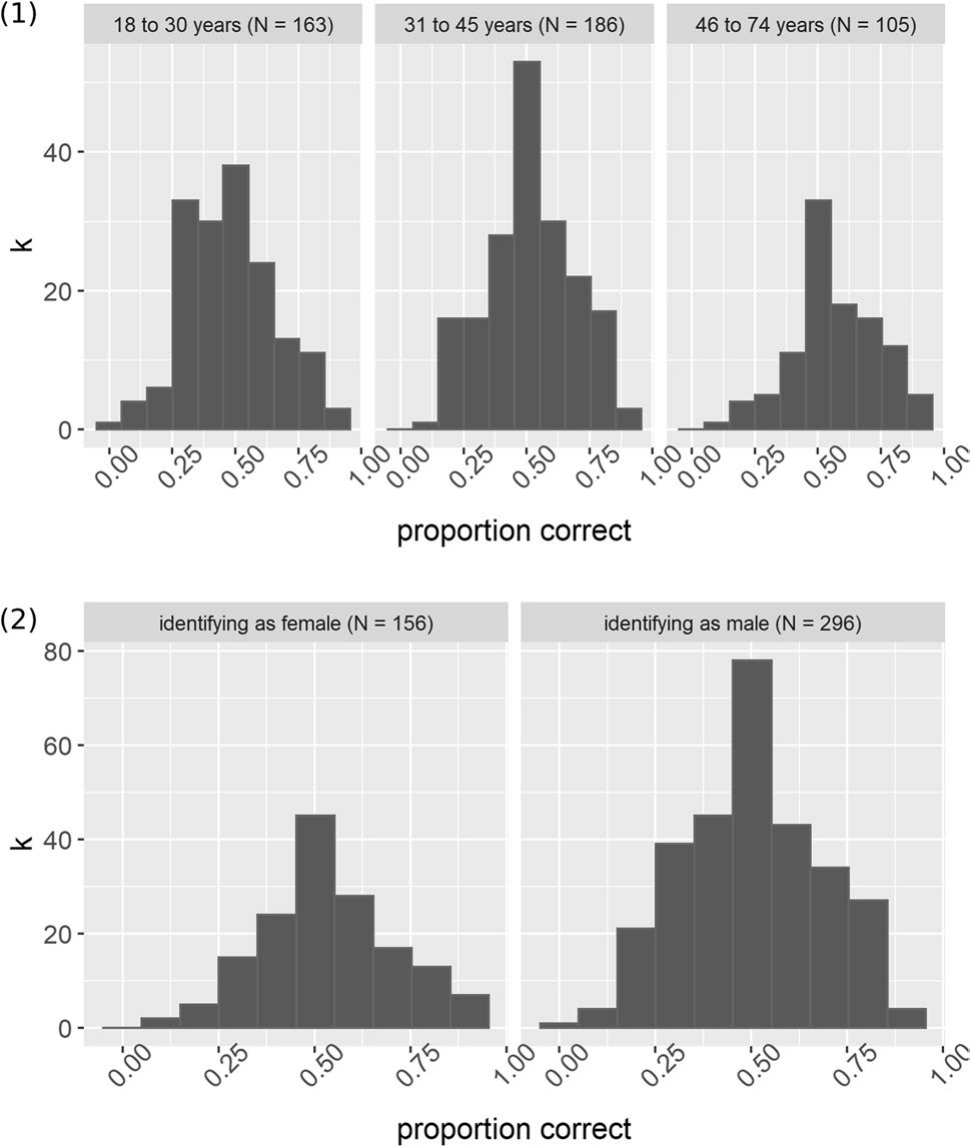
Test score distribution of the JVLMT divided by age groups (1) and gender (2)

*Gender and age.* We found a trend for a performance advantage of female (M_female_ = 0.54, SD = 0.17) vs. male (M_male_ = 0.50, SD = 0.18) participants (*t*[323] = 1.91, *p* = 0.057) in the JVLMT. By inspecting the means of the three age groups, it seemed surprising that young listeners had lower accuracies than old listeners, which is in contrast to previous findings (Zäske et al., 2018). We therefore tested for statistical differences in the accuracies between the young and old age group in our sample. A *t* test revealed that old listeners indeed had significantly higher accuracies than young listeners (*t*[226] = – 4.30, *p* < 0.001, Cohen’s *d* = 0.53; M_young_ = 0.48, SD_young_ = 0.17; M_old_ = 0.57, SD_old_ = 0.17). Table 2 and Fig. 7 give an overview of performance differences among participants of different genders and age groups.

**Table 2:** Descriptive statistics for JVLMT testing phase performances (proportion correct) in different groups. Combined norms for age and gender groups are provided along with the downloadable test in the supplemental materials (Table S10)

	Identifying as female (*n* = 156)	Identifying as male (*n* = 296)	Aged 18–31 years (*n* = 163)	Aged 31–45 years (*n* = 186)	Aged 46–74 years (*n* = 105)
M	0.54	0.50	0.48	0.51	0.57
SD	0.17	0.18	0.17	0.17	0.17
Range	0.11–0.91	0.05–0.95	0.05–0.91	0.13–0.91	0.12–0.95

*Phonagnosia and super recognition.* A total of *n* = 7 individuals with a potential phonagnosia as well as *n* = 4 individuals with potential super recognition abilities were identified (displaying mean performances at least 2 SDs below or above the mean performance). An overview of their individual performances in all four tests can be found in the supplementary materials (Table S7).

English Proficiency. Test scores of the JVLMT and the self-evaluated language proficiencies correlated only weakly (*r* = 0.24), indicating higher performance with increasing language proficiency.

## Discussion

We present a new standardized test to measure voice learning and memory abilities in adults within ~ 22 min, using complex utterances with speech-like phonetic variability (available online from: https://osf.io/cyr23/). The 22 test items of the Jena Voice Learning and Memory Test (JVLMT) were selected using acoustic similarity measures and IRT, such that items vary in difficulty within an intermediate range of the ability spectrum. It can further serve to screen for potential phonagnosia or super-recognition abilities, although clinical diagnoses should only be made following an in-depth examination, including comprehensive test batteries. The test uses pseudo-sentences based on a repertoire of English phonemes. By relying on sentences, rather than simpler utterances (such as vowels or syllables as used in previous tests), the JVLMT accounts for the complexity and phonetic variability of human speech. Please note that unlike previous tests, the JVLMT is designed to capture voice recognition skills regardless of whether the identifying features originate from aspects of voice quality (i.e., f0 and timbre), or from more coarse-grain spectro-temporal properties (such as idiosyncratic phonetic patterns or micro-hesitations), which can also contribute to speaker identification in the absence of voice quality (Remez et al., 1997). A degree of culture-fairness is achieved by the use of pseudo-speech devoid of meaning, such that interaction with semantic processing is precluded. At the same time, preliminary data suggest that overall performance is independent of language background and proficiency. The test is thus suitable for international research contexts. Complementary to existing tests, the JVLMT measures long-term voice memory abilities sensu current person perception models (Belin et al., 2004; Young et al., 2020), rather than short-term discrimination abilities (compare BVMT; Mühl et al., 2018). Accordingly, the JVLMT targets voice representations which store relatively abstract, speech-invariant voice properties, in the sense that voice memory is measured across study-to-test changes of speech content for a given speaker, rather than by means of specific vowel samples which are repeated at test (compare GVMT; Aglieri et al., 2017). As would be expected based on these conceptual differences between tests, the JVLMT therefore exhibits a substantial degree of convergent validity with the BVMT (Mühl et al., 2018), and a moderate degree of convergent validity with the GVMT (Aglieri et al., 2017). Discriminant validity with general working memory abilities is reflected in the finding that differences in JVLMT test scores are only weakly accounted for by general auditory working memory capacity (digit span). The JVLMT has an empirical reliability of 0.66, IRT-based. We anticipate that our test will facilitate research on individual differences in voice learning and memory, and will be a valuable tool to study cognitive and neuronal bases of these differences.

### Individual differences

The JVLMT is capable of determining individual differences in voice learning and memory across an intermediate ability spectrum. We developed the test using IRT such that the final version contains 22 items ranging from easy to difficult. This is well reflected in both, the distribution of test scores from the 454 participants in the validation phase (see Fig. 6) and in mean performances across items (see Fig. 5). While the great majority of test scores accumulate around the mean, test scores range from 0.05 to 0.95. This wide range of scores of the JVLMT is comparable with other voice perception and memory tests, i.e., the BVMT (Mühl et al., 2018) or the GVMT (Aglieri et al., 2017), and is also in line with large individual differences in the recognition of personally familiar voices (Skuk & Schweinberger, 2013).

At the low end of the ability spectrum, we identified seven individuals with at least 2 SDs below the mean, and four individuals performing at least 2 SDs above the mean at the high end. This could indicate individuals with possible phonagnosia and super-recognition skills, respectively. Note that while JVLMT performance differences between these subgroups also tend to be seen in convergent validation tests (see supplemental table S7), there are also possible cases in which poor JVLMT performance occurs in the context of preserved voice matching in the BVMT (e.g., case P271). Such a pattern of findings would be in line with both, the claim that voice discrimination (matching) and recognition are separate abilities (Van Lancker & Kreiman, 1986, 1987) and with neuropsychological cases #5, #24, and #30 reported by Neuner and Schweinberger (2000). At the same time, there are no obvious differences in digit span performance between those two subgroups, in line with the notion that voice memory recruits different brain areas and mechanisms than memory for other auditory objects, i.e., words or environmental sounds (Belin et al., 2000; Stevens, 2004). Of note, the JVLMT is best suited to measure voice memory abilities within a “normal” ability range and is less well suited to reliably assess outstandingly low or high levels of ability. Hence, while it can serve as an initial diagnostic screening tool for potential phonagnosia or super-recognition abilities, any such suspicions should be backed up by further tests. We envisage that future extensions of the JVLMT might include further modules and/or items to enhance assessment at the respective end of the performance spectrum.

On a group level, findings on the existence of gender differences in voice recognition are mixed, but when such differences are reported, women tend to outperform men (Skuk & Schweinberger, 2013; Roebuck & Wilding, 1993; Aglieri et al., 2017; Cortes et al., 2017; Bartholomeus, 1973; Thompson, 1985; Yarmey & Matthys, 1992). Somewhat in line with these observations, test scores in the JVLMT from female participants tended to be higher than those from male participants. While the cause of gender differences in voice memory has not been systematically studied yet, possible mechanisms could include increased social contact (and perceptual expertise with voices) in female listeners, attentional differences or differences in voice memory capacity. Of interest, older listeners outperformed young listeners in the JVLMT (see Table 2). This seems at variance with earlier findings from the laboratory and with meaningful sentences (Zäske et al., 2018). The reasons for this discrepancy are not entirely clear. We speculate that, in the context of the present online study, performance of older participants may have benefitted from a positive self-selection for proficient use of technology, higher levels of conscientiousness (Soto et al., 2011), more focused and attentive task performance, or a combination of these factors. Unfortunately, due to pandemic restrictions, our laboratory sample was too small to investigate these age-related differences in JVLMT test scores. Further research under laboratory conditions, with participants of different age groups and additional measures is needed to replicate and explain these age effects.

We also assessed a possible “language familiarity effect” (LFE), as another known factor in voice recognition performance (Fleming et al., 2014; Perrachione & Wong, 2007; Perrachione et al., 2011; Zarate et al., 2015). Accordingly, it might be suspected that the JVLMT disadvantages participants with relatively low perceptual expertise with spoken English, as our pseudo-sentence material is based on English phonology. However, the present data from English-speaking participants do not point to strong effects of language background of our listeners. The correlation between the JVLMT test scores and the participants’ self-evaluated language proficiency based on the Common European Framework of Reference for Languages (Council of Europe, Modern Languages Division, 2001) was only weakly positive, suggesting that our careful stimulus development resulted in fairly LFE-free items. As there are several studies indicating only weak or moderate correlations between subjective and actual measures of language skills (Edele et al., 2015; Li & Zhang, 2021), we also analyzed effects of participants’ native language on test performance, as a more objective measure of language proficiency. According to these analyses, native language did not seem to be a significant factor in explaining the results obtained for the item selection sample and validation sample. We therefore propose that the JVLMT can be used to assess voice recognition within English-speaking participants, although it needs to be kept in mind that the present sample only included a relatively small and heterogeneous sample of non-native English-speaking participants. Accordingly, due caution is necessary regarding this issue until further data with larger, representative listener samples is available.

Regarding test fairness across different groups of participants, testing differential item functioning (DIF) provided evidence that the test is fair with respect to gender (female vs. male) and age (≤ 35 vs. ≥ 36 years). In other words, our items measure the same underlying ability across these groups and increasing item difficulties correspond to increasing ability levels within each group. Note that test fairness is independent of the fact that, on average, test scores may differ as a function of age and gender (as discussed above). Regarding culture fairness, it remains to be established if the JVLMT measures the same voice processing abilities across people of various ethnical or language backgrounds. In that regard, and considering previous research on LFEs, we recommend that future studies should more systematically assess test fairness of the JVLMT within (and performance differences between) major groups with different language backgrounds, and then assess DIF with respect to these groups.

Among other factors that could contribute to individual differences in voice learning and recognition, autism may compromise voice identity processing in some situations (Schelinski et al., 2017) but not others (Lin et al., 2015). High levels of autistic traits in the neurotypical population can be associated with poor recognition of personally familiar voices (Skuk et al., 2019). Note that previous research including the above studies, used “in-house” developed voice recognition tasks for lack of standardized alternatives, thus complicating the interpretation of conflicting results. The JVLMT now provides the opportunity to test participants from the autistic spectrum with the same tool across labs to make results more easily comparable and interpretable.

Finally, research on a possible role of auditory expertise on individual differences in voice recognition is based on blind listeners, musical experts and multilingual individuals, but results are scarce and heterogeneous (Föcker et al., 2012; Hölig et al., 2014; Bull et al., 1983; Pang et al., 2020; Eladd et al., 1998; Gougoux et al., 2009; Guenzburger et al., 1987; Winograd et al., 1984; Chartrand & Belin, 2006; Xie & Myers, 2015; Levi, 2018 and Theodore & Flanagan, 2020). We anticipate that it will be beneficial to use the JVLMT to assess voice recognition in these groups.

### Applications and limitations

To the best of our knowledge, the JVLMT is the only standardized tool that assesses voice learning and recognition that generalizes across previously unheard and complex utterances. Moreover, the JVLMT has been developed using the latest test theoretical standards (IRT) and can be used on English-speaking participants or patients. With a testing time of ~ 22 min, the JVLMT is time-efficient and can be easily integrated in research projects investigating voice processing abilities among the general population and how these abilities are linked with other aspects of perception and cognition. Prospectively, it can also be used as a screening tool in clinical settings to help detect voice recognition impairments in neurological (Neuner & Schweinberger, 2000), or psychiatric patients (Stevenage, 2018), particularly in autism (Schelinski et al., 2017), or other conditions in which voice recognition difficulties may be more prevalent without being routinely screened for (Neuner & Schweinberger, 2000).

The JVLMT is the first instrument to measure voice learning and recognition, conceptualized as the successful acquisition of abstract speech-invariant voice representations. Although the test taps into long-term memory (in the range of at least a few minutes) the degree to which voice representations acquired during learning are retained over longer periods of days or weeks, remains unclear, both for the JVLMT and the GVMT (Aglieri et al., 2017). The longevity of voice memory following these tests should be determined and compared in future studies, in order to assess the time which needs to elapse before these tests can be re-administered. In a similar vein, it would be interesting to investigate the link between memory abilities for newly learned voices in the JVLMT with the ability to recognize personally familiar voices. The process of learning a new speaker in the JVLMT does not necessarily reflect how we acquire new voices in everyday interactions in which we are typically also exposed to peoples’ faces or other cues to identity. While the JVLMT was designed to target the auditory modality, in light of known interactions between voice and face processing systems (Young et al., 2020), it would be a logical next step to develop a multimodal voice learning and memory test, as has been done for vocal emotion recognition (e.g., Bänziger et al., 2009, 2012) and personally familiar (famous) people (Quaranta et al., 2016). Considering that voice learning in everyday-life may often proceed automatically and non-intentionally, one should be aware that the JVLMT (like other tests on person memory), arguably captures a particular kind of learning. Specifically, by asking participants to remember voices for an upcoming test, the JVLMT taps into intentional learning, which has been associated with higher performance rates and relies on partially different neural mechanisms than incidental voice learning (Humble et al., 2019).

Furthermore, the stimuli developed for the current work may not contain the range of variability, typical for natural social encounters where prosody and emotional expression can change rapidly and frequently. Lavan et al. (2019) point out further, that highly standardized stimuli in laboratory studies compromise this natural within-person variability. This within-person variability could contribute to voice learning because recognizing a speaker means generalizing across within-person variability (“telling people together”) and “tell people apart” on the basis of between-person variability. Although the present stimuli were standardized to a fair extent and thus did not contain the entire range of within-speaker variability possible, the fact that we used several sentences rather than vowels can be considered an advantage in terms of ecological validity, compared to the GVMT as the only other existing voice learning test (Aglieri et al., 2017).

With a testing time of ~ 22 min, the JVLMT may be considered a relatively extensive test compared to the GVMT (~ 5 min). However, this extra time seems well invested if one wishes to measure voice learning and recognition across a variety of speech-like utterances, in order to capture the formation of idiosyncratic and abstract representations of vocal patterns. The marginal (empirical) reliability of the JVLMT is 0.66, and represents the mean reliability of all items. An advantage of tests constructed using IRT is that reliability can be determined for individual items. Therefore, we additionally assessed the overall measurement precision with the test information function or standard errors of test scores (as depicted in Fig. 4, panel 2) as this provides a more fine-grained evaluation of measurement precision at any point of the ability scale. As depicted in this figure, the measurement precision is quite high over a broad area of ability levels and especially around the midpoint of the scale but decreases towards the more extreme ends of the scale. Because the JVLMT measures most reliably in the intermediate ability spectrum, it is best suitable to quantify voice recognition abilities in the “normal” population. As a future project to render the JVLMT a more sensitive diagnostic tool, that discriminates well across the entire spectrum of abilities, further items, or optional extension modules, could focus on item difficulties adapted to participants with low and high ability levels, respectively. At present, lower or higher scores in the JVLMT should be interpreted with caution and warrant further tests to confirm suspicions of phonagnosia or super-recognition abilities. For clinicians or users looking for super-recognizers, the JVLMT can serve as an initial screening tool, which we recommend using in the context of more extensive test protocols. While we expect that further voice tests will be available in the near future, it should be our aim to introduce common diagnostic standards and test batteries, equivalent to current efforts in face research (e.g., Ramon, 2021).

### Future directions

With a standardized test such as the JVLMT it is possible to accomplish a more systematic understanding of voice learning and memory. Our sample provides norms for neurotypical adults, however, it would be helpful to norm it for neuropsychological patients as well. Apart from patient-specific norm data, the test might be extended by modules for super recognition and phonagnosia, or by a version for children (compare analogous developments for the CFMT in the face domain, Croydon et al., 2014). An essential characteristic of a good test is also a continuous assessment of its quality criteria, such as reliability and validity. To keep the JVLMT up-to-date, it needs to be ensured that this assessment is accomplished to confirm its functionality regularly (Kersting, 2007).

According to the revised functional model of face and voice perception (Young et al., 2020), voice perception contributes less to identity analysis than does face perception. Therefore, the development of a multimodal learning and memory test might be the next step to assess how humans differ with respect to identity learning and memory. Comparing these test scores with performance from unimodal person perception tests would allow for a detailed assessment of person perception skills on an individual level. Adding neuroscientific methods such as EEG and fMRI could then complete the picture by providing information about the individual functioning of the systems for processing person identity.

Finally, we would like to note that this is the first study linking the location of individual voices in an acoustic multidimensional voice space (MDVS) to recognition performance following learning. Specifically, participants’ recognition performance in the item selection version of the JVLMT was significantly improved when three test voices on a given trial were acoustically dissimilar (i.e., situated far apart from one another in the MDVS) compared to acoustically similar voices (situated in close proximity in the MVDS). This lends further support to the notion that the MDVS framework is suitable to model important phenomena of voice memory and provides evidence for a link between physical voice properties and perception. Our finding is particularly remarkable, considering that it was obtained for complex pseudo-sentence stimuli (vs. vowels or syllables as seen in previous works by Baumann & Belin, 2010; Latinus et al., 2013; Mühl et al., 2018), and using the same acoustic parameters (f0, FD, HNR) which also define the voice space for vowels (Latinus et al., 2013). For more complex stimuli, such as the present pseudo-sentences, it remains to be determined whether the inclusion of additional parameters that capture prosodic or temporal characteristics can increase the prediction of memory performance based on individual voice location in MDVS.

### Conclusions & Outlook

Individual differences in voice learning and memory have usually been assessed using “in-house” developed non-standardized tests with a focus on identifying possible impairments (e.g., Neuner & Schweinberger, 2000; Roswandowitz et al., 2014; Van Lancker & Kreiman, 1986, 1987). However, the ability spectrum for these skills is broad and only very recently have researchers begun to develop standardized tests to assess individual differences in healthy populations (Aglieri et al., 2017; Mühl et al., 2018). The JVLMT as described here has been developed on the premise to reflect an intermediate ability spectrum with the option to use it as an initial screening tool for exceptional voice memory abilities (phonagnosia or super-recognition). By combining the JVLMT with assessments of other person perception skills, individual differences can be examined and more easily compared between labs worldwide. By integrating the JVLMT into screening procedures of neuropsychological or hearing-impaired patients, it could find its way into clinical practice and help to inform about impairments in person perception.

In conclusion, the JVLMT is an efficient and freely available computer-based assessment tool with the potential to facilitate research into voice recognition and the cognitive and neural mechanisms underlying individual differences in person perception.

## Supplementary Information

Below is the link to the electronic supplementary material.

Supplementary file1 (DOCX 14 KB)

## Footnotes

1 Because “the” was the only article, five personal pronouns were added to this word category to introduce more phonetic diversity (cf. supplementary materials).

2 The complete R script for this selection process is available upon request from the corresponding author.

3 Note that another minor difference between the first and second learning-test-cycle was the timing of the familiarization trials. Specifically, the blank screen preceding the prompt “new speaker” lasted 1500 ms in the first block, and 500 ms in the second block.

4 Note, that we preferred these parsimonious models over more complex models as a starting point, in order to obtain a sum score for the test which would be readily interpretable for the convenience of users. By contrast, a more complex 3PL model, for instance, would require additional tools to interpret the resulting scores, depending on the exact response pattern in the test. However, because the additional guessing parameter in the 3PL would account for an important source of individual variability and would be desirable from a theoretical test construction point of view, we also tried to assess a 3PL model. However, the 3PL model could not be estimated due to empirical non-identification.

5 Please note: *n* = 40 participants did not meet the criterion for the minimum digit span length of four in our test battery because they failed to recall at least five items with a sequence length of four digits correctly. Not to lose the data of these 40 participants for the correlation analyses we decided to replace their missing values with 2.5 which represents the mean of the two shortest sequence lengths (of two and three digits) which were, however, not implemented in our test battery. The correlation was also calculated without these 40 participants (*r* = 0.10).
