# Supplementary material for: The Jena Voice Learning and Memory Test (JVLMT): A standardized tool for assessing the ability to learn and recognize voices
Source: Behav Res Methods. 2022 Jun 1;55(3):1352–71. doi: 10.3758/s13428-022-01818-3 (PMC10126074; doi:10.3758/s13428-022-01818-3)
Supplement: Supplementary file 1 — Supplementary file1 (DOCX 14 KB) [file 13428_2022_1818_MOESM1_ESM.docx]

**Supplementary Materials**

Follow this link to the supplementary materials, user instructions and the downloadable JVLMT as programmed in PsychoPy:

https://osf.io/cyr23/

**Data and Analysis Scripts**

For review purposes, please follow this temporary link to data and analysis scripts (R-based):

https://osf.io/u2fjq/?view_only=6fc4684f264f481ead9272245107fa70

The folder “open-access-scripts” contains 3 subfolders with analyses from pertaining to:

1. Main manuscript, section 2.5.4.1 Item Selection
2. Main manuscript, section 2.5.4.2 Behavioral Results
3. Supplemental Materials on General logistic mixed model analyses (glmm)
   1. Section 2.3 glmm on item selection sample
   2. Section 2.3 glmm on validation sample

Upon acceptance these data will be made public and permanent on https://osf.io/cyr23/
